# Notes of an European Tour

**Published:** 1846-04-01

**Authors:** F. H. Hamilton

**Affiliations:** Professor of Surgery in Geneva Medical College


### BUFFALO MEDICAL JOURNAL

**Vol. I.** APRIL 1, 1846. **No. 11.**

### Notes of an European Tour.---No. 11.

Communicated for the Buffalo Medical Journal, by F. H. HAMILTON, M. D., Professor of Surgery in Geneva Medical College.

PALERMO, SICILY.

Palermo has a population of nearly 200,000, most of whom retain in their features and complexion evidence of their mingled Greek and Asiatic origin;—their manners, customs and style of architecture are also strikingly Saracenic.

The streets are, with few exceptions narrow; the buildings lofty, the upper stories of which are furnished with light balconies, or with more heavily constructed stone galleries, shut in by a strong iron grating, the latter indicating a nunery or the monastic prison of some religious order. The small ground rooms are chiefly occupied as shops, in front of which sits at his daily work, the tailor and his goose, the cobler and various other artisans,—with the vender of trifles, at whose stall, generally placed near the door of a church, you may purchase wax tapers and other votive offerings, amulets, painted wood cuts of the Virgin Mary, and of all the saints, from Santa Apollonia, who presides over the toothache, and St. Hyacynth, who insures against sterility, down to St. Martin, who cures "scabies," and St. Fiage, who keeps a "lock hospital." Here you will find also paste and bead rosaries, pewter crosses, gilded wooden images of our Saviour, red, black and white coral, beautiful Sicilian agates, chrystals of sulphur, and rare Sicilian shells. If you walk the Strada Toledo about four hours past midday,, you will be compelled to thread your way among these various occupants of the road; you will be jostled also by banditti looking soldiers, brushed by priests, and pursued by a legeon of beggars. Alas for Sicily—her fertile soil and luxurious climate have long rendered her the miserable prey of her more powerful neighbors, and the land once named the "Granary of Rome;" is now overrun with rank weeds and nourishes only priests, soldiers, robbers, assassins and lazzarones. The commerce of Palermo, once extensive in corn, cattle, wines, &c., is now dwindled into a petty trafic in silks, oranges, lemons, sulphur, soda, manna, saffron, cork, sumach, &c. The spirit of the people naturally bold and impatient, flaming forth every now and then, despite a strong military force, into the most terrific and bloody insurrections, bends tamely to a most intolerant religious despotism; and instead of occupying the summit of Monte Pelligrino—a natural and impregnable fortress for the city, and which Hamilcar Barcas, the Carthagenian general gallantly defended for more than three years—with arms and men of steel; it is occupied by confessionals, and defended by broken wax legs and wax arms, and a score of stupid priests. Education extends rarely beyond learning to read, and an accomplished lady entertains her friends with specimens of her chirography as evidence of her advance in science, and with an embroidered "bambino," as proof of her skill in the fine arts.

It cannot be supposed that in such a country the science of medicine has made much progress.—Medical students go abroad to receive their education, and although several physicians and surgeons in Palermo deservedly hold a high rank, yet they complain of the successful rivalry of the Priests and the "Salassatori." The Priests, or what is equivalent, his "holy relic" often obtains the credit of the cure, even when a regular physician is employed. The "Salassatori" are found in almost every street; the shops being indicated by a barber's pole, two large copper basins and a horse tail; occasionally, also by a vile painting, representing a "Seneca," throwing blood like a jet d'eau from a dozen orifices. Within, is a swarthy Sicilian, who will furnish you salves for ulcers, cancers and tumors, will leech and pull teeth, will bind up your wounds and mend your bones, will bleed you by the ounce, will shave, cut hair and point your "imperial." These are the veritable representatives of the ancient "Barber Surgeons," whose "ensign," in the twelfth century was a pole wrapt with a red roller, supported by two basins; of which honorable fraternity the great Pare boasted himself a member, and from which the present "royal stock" of surgeons are lineal descendants. It is therefore that I have examined the more in detail these establishments, one of which I entered and explored entirely to my satisfaction; which done, I requested the surgeon to bleed me. 'How many ounces Signore?' 'Six.' 'Where?' 'In the arm.' Immediately I was divested of my coat—my hand was made to grasp the top of an upright rod, supported by three legs—my sleeve was turned up smoothly and tenderly above the elbow—the blood red fillet was then applied in a most artistical manner, a spear pointed lancet selected from the arsenal, and already was the thirsty weapon glittering in the air, when I withdrew my arm, and declared myself satisfied. It was as a *pupil* and not as a patient, that I had entered the office of this descendant of my Fathers. Francesco paid him the two carlini, and we went on.

Homœopathy has also lately attracted a large share of patronage; and in Palermo as at home, its converts are chiefly among the most aristocratic circles, over whom such sugared pretences have always a remarkable power of fascination; and in Sicily, Homœopathy now threatens fairly to supplant the sister faith in amulets, and the bottled breath of departed saints!

But Palermo has upon its civic record one name too distinguished in the annals of our science to be omitted. John Philips Ingrassias, a Sicilian by birth, and a citizen of Palermo, was in 1563 made "Physician in chief" to Sicily, with power to regulate and control the entire medical police of the country. Through his prudent instructions and skillful management, a fearful plague was arrested in the year 1575: in gratitude for which the Palermitan authorities offered him a liberal reward, of which he accepted only one half, and this also he immediately devoted to religious purposes. Ingrassias was an acute Anatomist, and his minute dissection and acurate description of the different parts of the base of the brain has attached his name to one of its bones; a singular but touching memorial of his scientific and humane labors, which has secured to him a perpetual tablet in this "Dome of thought and palace of the soul:" He died in 1580, at the age of 70, honored and regretted by his fellow-citizens.

About two miles out of Palermo, at the foot of Monte Griffone, is a large cavern filled with petrified bones; and which having given rise to much speculation, I felt anxious to visit. When first opened in 1830, it was rumored that the sepulchre of the ancient Cyclops had been found—those huge giants, whom Æneas and his crew are fabled to have seen upon these shores,—

> "A dread assemblage! with their heads on high,
> Not yielding to the towering tree of Jove,
> Or tallest Cypress of Diana's grove."

But they prove to be skeletons of elephants, hippopotami, crocodiles and a great variety of other land and aquatic animals; some of which are not now found in Sicily, and others are thought by certain antiquarians to be antediluvian. The latter supposition is however, not at all probable, since they are found only in a bed of limestone, formed evidently by a gradual deposit from the springs above, and if the skeleton of a mammoth has been found, as is asserted, it must have been brought here at the same time with the other bones, which are clearly post-diluvian.

Furnished with torches, hammers and baskets, we were enabled in an hour to collect a considerable quantity of these remains, which proved to be the tarsal and meta-tarsal bones of some animal to me unknown; large molars, probably of oxen, femurs, &c. No human bones have been found, and the most reasonable supposition is, that this was a burial place for such animals as died from an ancient and extensive menagerie, or naumachia.

Returning we overtook a drove of Sicilian oxen and cows—they were uniformly of a dun color, and larger and more beautifully formed than any I ever saw—their horns also were enormous in length and size.—When Apollo was a farmer, these were his favorite cattle, and "on sols bright Isle, Trinacria,"—(Sicily) he chose their pasture.

May 30.—I have seen a gentleman direct from Malta, where, although he sailed from Alexandria with a "clean bill," he has been detained several days. While in quarantine, he learned that since he left Alexandria, the plague had broken out, and now it will be impossible for travellers returning from Egypt to escape a prolonged detention at Malta, one, or two months! This news is fatal to my project of visiting Egypt, and I have reluctantly decided to steer again to the North—"Search Italy, since Jove denies me Crete."

NAPLES, JUNE 2.

I left Palermo in a Sicilian steamer bound for Naples, on board of which I became acquainted with Mr. Burchard of Hamburg, Germany, who speaks English and Italian, with almost equal facility with his native tongue. I find him a most agreeable as well as useful companion. We have taken a suit of rooms on the "Chiaja" together, and after spending a few days at Naples we shall journey by vetturino to Rome. * * * * * * * * *

"*Vedi Napoli epo Mori.*"—I came to Naples, a sceptic in relation to the charms of this famous bay and city, believing fully that travellers had written under the influence of strong classic associations, and a too sanguine sun: But my infidelity has ceased. No one can see the bay of Naples and its beautiful environs, without unmixed admiration—no one has ever described it as it is seen! Forgive me then if I draw for you but a rude outline map, a rough cartoon picture; the pencil of a Salvator Rosa alone can give the coloring.

Entering the bay from seaward, we pass on the right, the Island of Capri, and a long line of lofty and rugged rocks, extending from the main land to terminate in the promontory of Minerva; on the left, we coast close within shore, the Islands of Ischia, Procida, and Niseta, with the jutting promontories of Miseno and Posilipo; beyond which the city of Naples, reaching upward from the bay to the bending summit of Capodimonte, rises like a vast and gorgeous amphitheatre. Carrying the eye along the concave shore, until it rests over the prow of the vessel, a broad and conical mountain lifts itself from the waters edge; above whose blackened crest a dense column of smoke sways heavily in the breeze. On each side of Vesuvius, as if to bring into vigorous contrast the extremes of nature, stretch far back to the dim outlines of the Appenines, the fertile plains of the Campagnia—through whose open vales you catch at evening the first short breathings of the land-breeze, cool and refreshing and laden with the odors of vineyards and orange groves. Around the base of the mountain, and extending along the coast to the right, stand in quick succession the towns of Barra, Portici, Resina, Torre del Greco, Annunziata, Castel-a-mare, and Sorrento:—farther up, the slopes are studded with villas, and on every side their cragged summits are crowned with convents, castles, fortresses, and ruined temples. Like a mirror, within this carved and gilded margin, sleeps the bay; reflecting alike from its polished surface the bright and party-colored domes of Naples, and the gloomy turrets of the castle of St. Elmo—the green hill side, the mountain and the rock.

